# Surgical management of odontogenic myxoma: a case report and review of the literature

**DOI:** 10.1186/1756-0500-7-214

**Published:** 2014-04-05

**Authors:** Yoko Kawase-Koga, Hideto Saijo, Kazuhito Hoshi, Tsuyoshi Takato, Yoshiyuki Mori

**Affiliations:** 1 Department of Oral and Maxillofacial Surgery, Dentistry and Orthodontics, The University of Tokyo Hospital, 7-3-1 Hongo, Bunkyo-ku, Tokyo 113-8655, Japan

**Keywords:** Myxoma, Mandible, Conservative treatment, Long-term follow-up

## Abstract

**Background:**

Odontogenic myxoma is a benign odontogenic tumor with locally aggressive behavior, and is relatively rare in the oral cavity. There are currently no clear surgical management guidelines for odontogenic myxoma, and a variety of approaches may be used. This study evaluated the literature concerning the surgical management of odontogenic myxoma, and reports the long-term outcome of a case managed by using a more conservative surgical approach.

**Case presentation:**

We managed a 40-year-old Japanese man with odontogenic myxoma in the right mandible by enucleation and curettage, a relatively conservative approach that has proved to have been justified by a lack of recurrence over 10 years. Our strategy was compared with others reported in the literature, which was identified by a PubMed search using the term “odontogenic myxoma”. Articles without full text or with missing data were excluded. The age and sex of patients, the tumor location (maxilla/mandible), treatment (conservative/radical), recurrence, and follow-up period were compared in the reported cases that we evaluated. From the initial 211 studies identified, 20 studies qualified as mandibular cases of odontogenic myxoma. Recurrence was reported in three cases that had been treated with a more conservative surgical approach.

**Conclusions:**

Enucleation and curettage has proved an effective approach in several cases in ours there has been no recurrence more than 10 years after surgery but the risk of recurrence appears to be higher. We discuss the important factors that must be considered when determining the correct management approach to odontogenic myxoma.

## Background

Odontogenic myxoma is a very rare benign tumor that may arise in the maxilla or mandible, but which can be locally aggressive. It accounts for 3–6% of all odontogenic tumors [1,2]. Odontogenic myxoma is usually asymptomatic and is found incidentally on radiographs, appearing as a “soap bubble”. The lesions are not encapsulated, allowing substantial infiltration into the adjacent medullary bone. Consequently, odontogenic myxoma is generally managed surgically; however, there has been some debate as to the most appropriate surgical approach.

Reports of surgical treatment of odontogenic myxoma vary from simple enucleation and curettage to segmental resection and hemimandibulectomy. Recurrence rates are reportedly high, at around 25%, especially when a more conservative approach is taken [3]. Nonetheless, a more conservative approach exemplified by enucleation and curettagehas several advantages over more radical treatments like segmental mandibulectomy and mandibular reconstruction with fibular microsurgical flap formation [3]. There are currently no clear evidence-based surgical management guidelines for odontogenic myxoma.

Here, we describe the long-term outcome of a case of mandibular odontogenic myxoma managed by enucleation and curettage, in the context of a systematic review of the literature, focusing especially on recurrence.

## Case presentation

**Figure 1:**
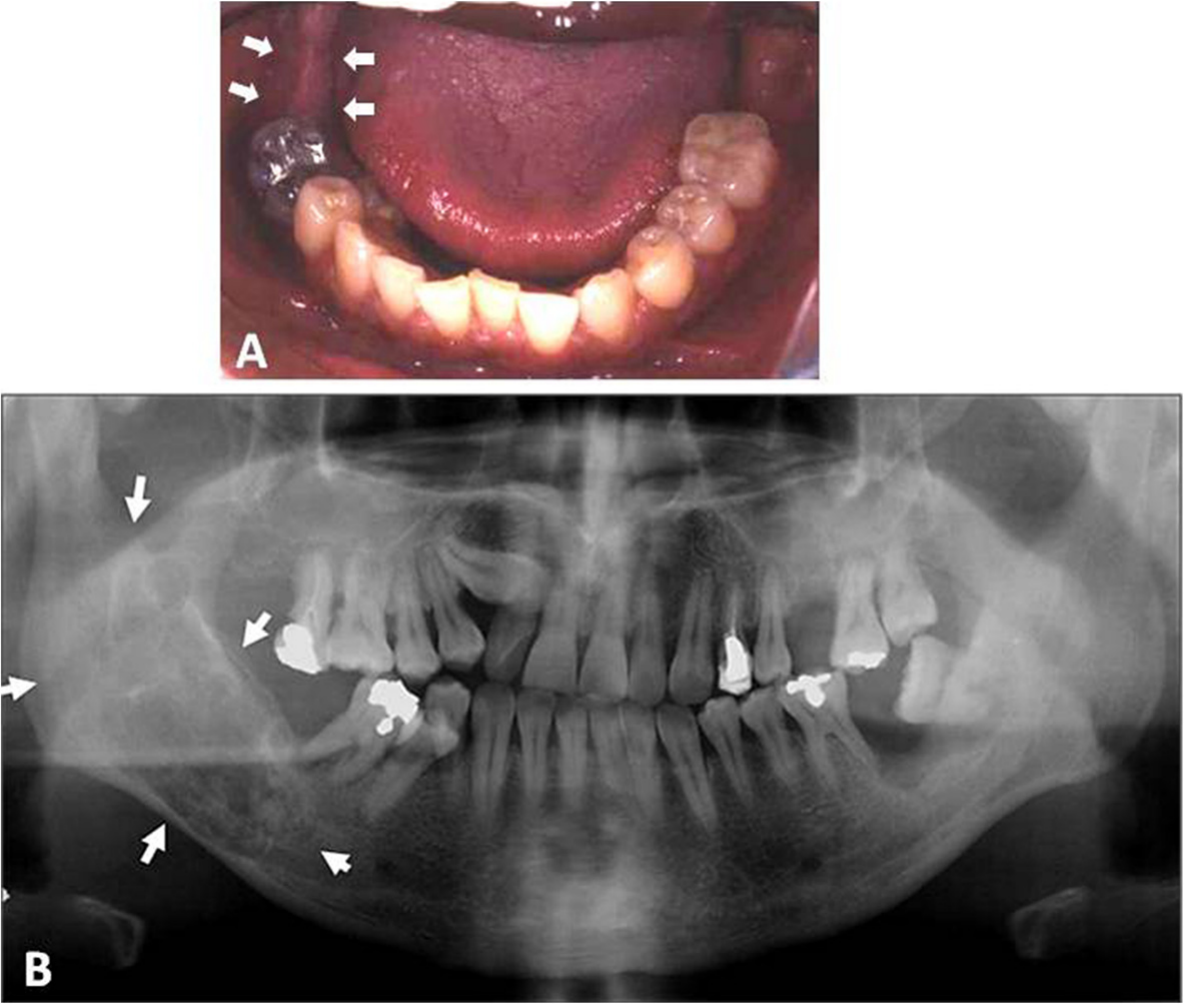
**Pre-operative findings: (A) Intraoral photograph showing no swelling (arrows). (B)** Panoramic radiograph indicating a “soap-bubble” appearance of the right jaw (arrows).

**Figure 2:**
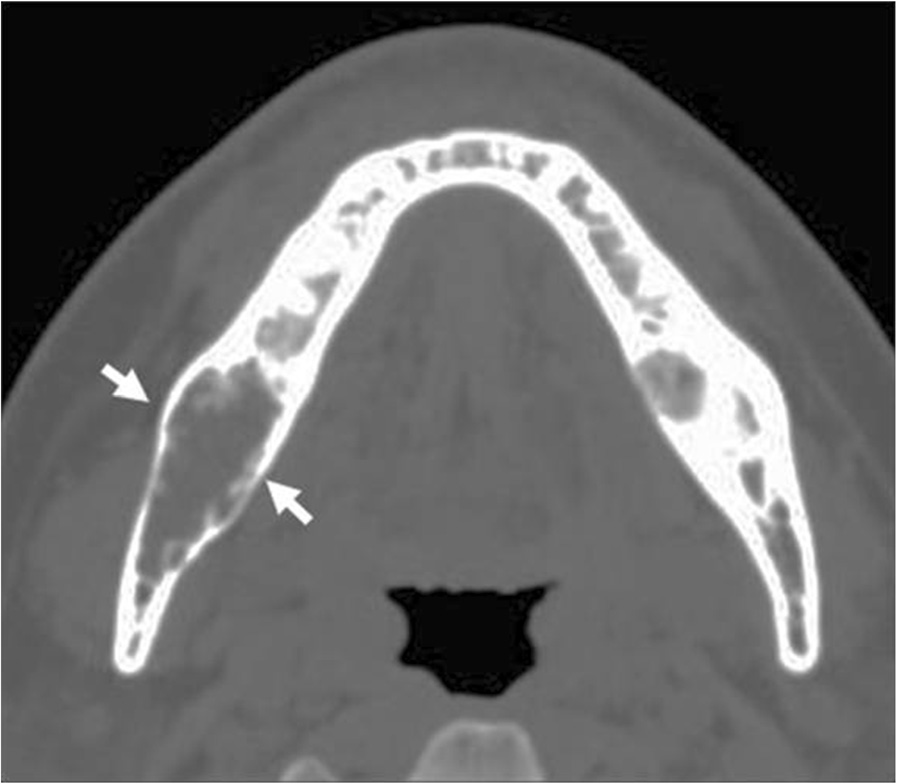
An axial computed tomography scan indicating a lesion on the right side of the mandible with thin residual bony trabeculae, and expansile invasion (arrows).

A 40-year-old Japanese man was referred to the Department of Oral-Maxillofacial Surgery, Dentistry and Orthodontics, at The University of Tokyo Hospital by a dental clinic after identification of a radiolucent finding on the right side of his mandible in December 2002. The patient reported no symptoms in his mouth including the mandibular area, and on clinical examination no swelling could be detected on the right side of the jaw, and the oral mucosa appeared normal (Figure 1A). However, a panoramic radiograph revealed an extensive radiolucent and multilocular area with imprecise borders that extended from the right posterior mandibular body to around the root of tooth #46, and exhibited a “soap bubble” appearance (Figure 1B). Computed axial tomography imaging showed an area of infiltration in the medullary bone with thin trabeculae in the right side of the mandible (Figure 2). The tumor measured approximately 30 × 15 × 40 mm. The patient’s medical history was otherwise unremarkable. An incisional biopsy showed loosely arranged spindle-shaped cells in a myxoid fibrous stroma. On the basis of these histological findings, a provisional diagnosis of odontogenic myxoma was made.

**Figure 3:**
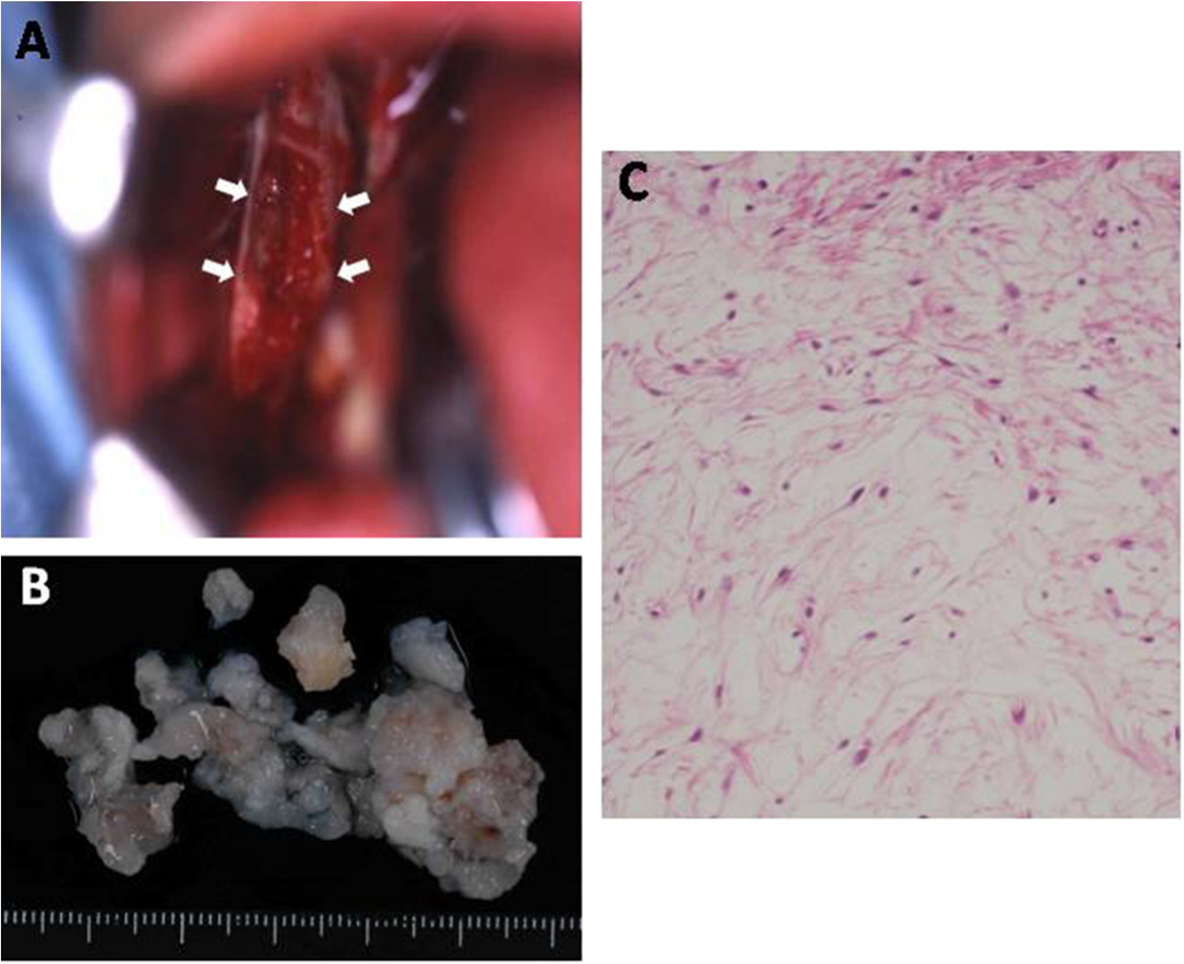
**Intra-operative findings: (A) Intraoral photograph indicating the lesion (arrows). (B)** Surgical specimen of the right mandible. **(C)** High-power view of histopathological findings.

**Figure 4:**
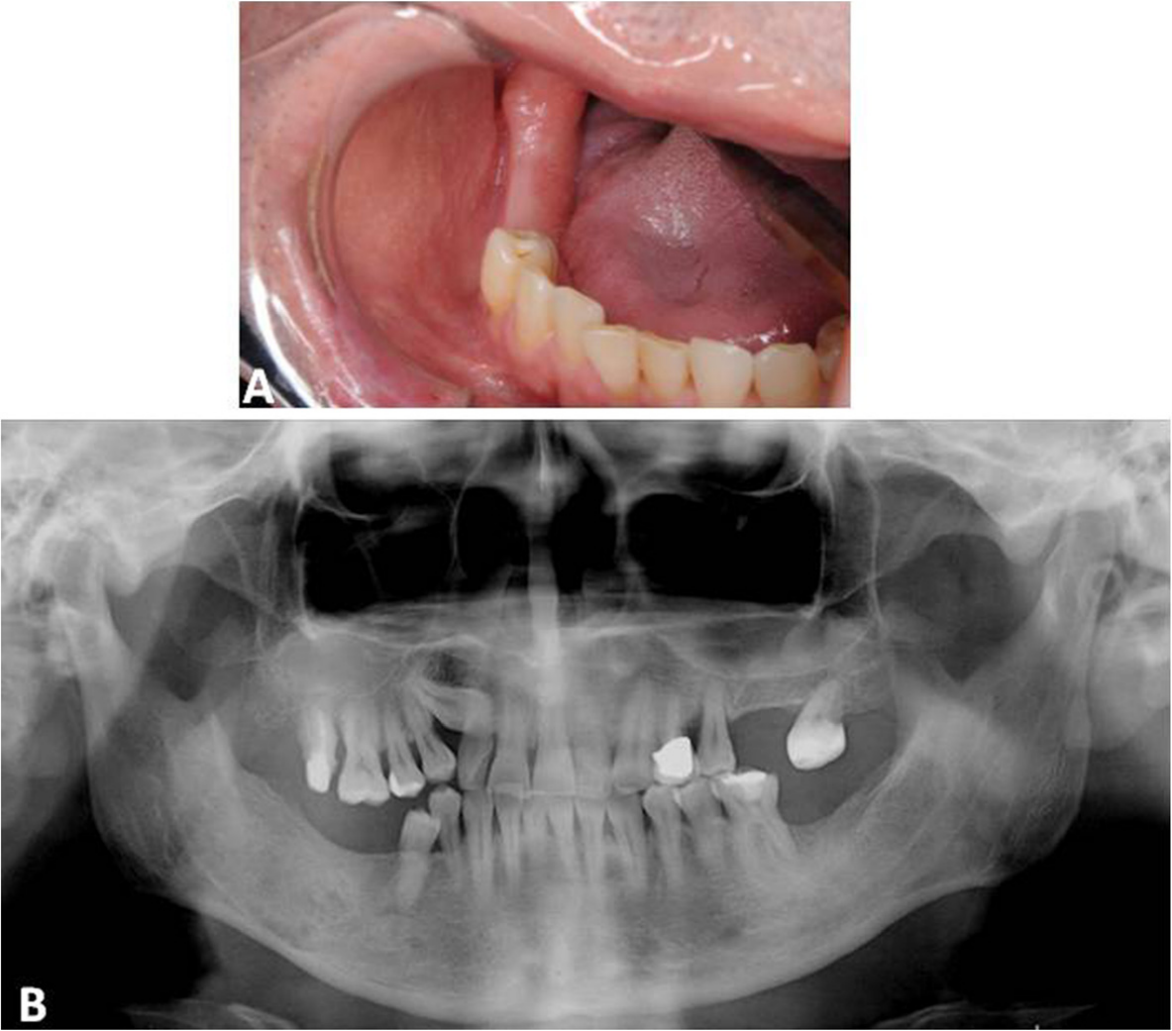
Post-operative findings: Intraoral photograph (A) and panoramic radiograph taken 10 years after surgery indicates full healing without recurrence.

We performed extraction of tooth #46, and an enucleation and wide curettage of the normal surrounding tissue to preserve the inferior alveolar nerve, the jaw, and oral function under general anesthesia with nasopharyngeal intubation (Figure 3A). The surgical specimen revealed benign-looking spindled and stellate cells in the mucinous stroma (Figure 3B, C). Taken together, these findings confirmed the diagnosis of odontogenic myxoma. There have been no clinical or radiological signs of recurrence over 10 years follow-up (Figure 4A, B).

### Systematic review of the literature

**Figure 5:**
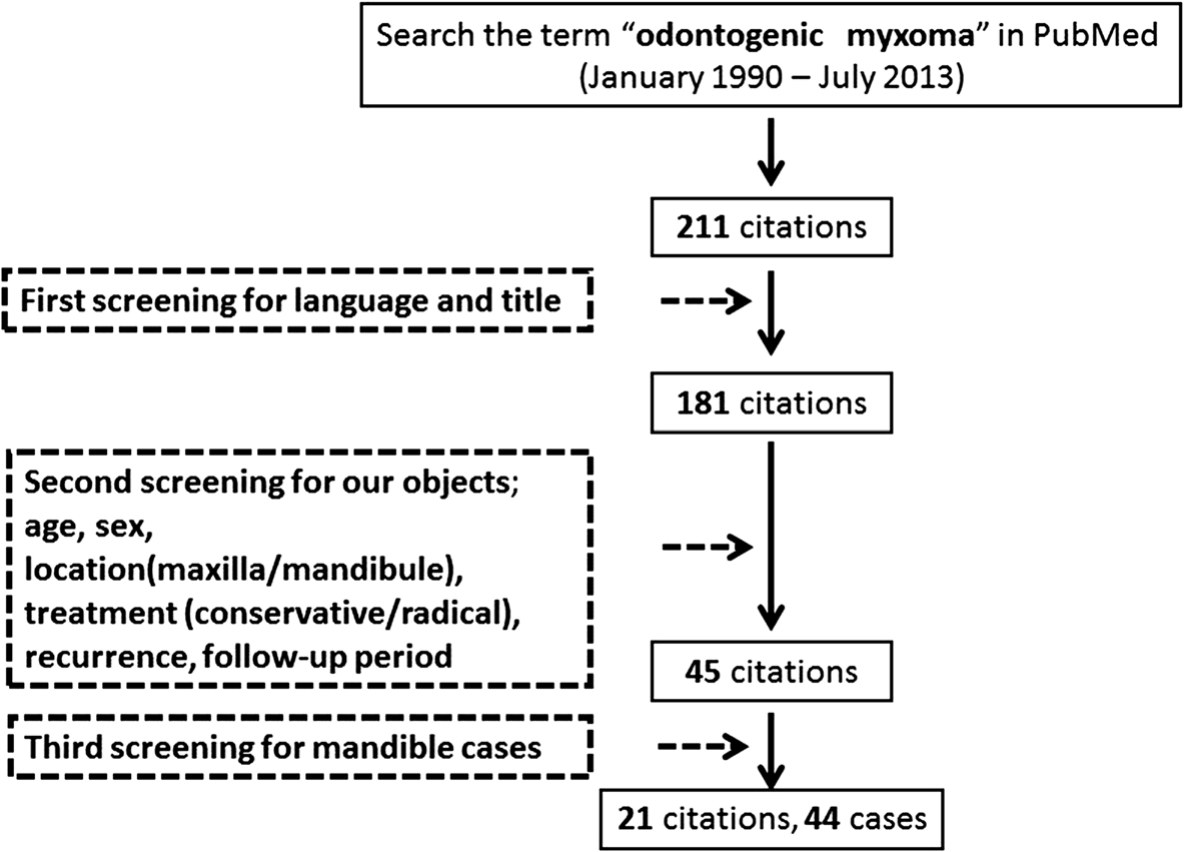
Systematic search strategy used to identify reports of “odontogenic myxoma” in the literature.

To investigate the influence of a conservative or radical surgical approach to odontogenic myxoma, we undertook a search of the PubMed database in July 2013 using the search term “odontogenic myxoma” that identified 211 studies published between December 1990 and July 2013 (Figure 5). Articles for which full text was not available or with missing data were excluded. The second step involved filtering the remaining 181 studies by patient age and sex, tumor location (maxilla/mandible), treatment approach (conservative/radical), recurrence, and follow-up period. Conservative treatment was defined as enucleation, curettage, and marginal resection; radical treatment was defined as segmental or block resection, and hemimandibulectomy requiring reconstruction. After this second step, 45 articles were identified, of which 21 (reporting 44 cases of mandibular disease) were selected for analysis.

Details of the 44 cases are given in Table 1. The mean age of patients was 31.9 years, and 22 (50.0%) were male. Twenty of the 44 cases (45.5%) were managed by conservative surgical techniques, the remainder underwent radical treatment. Tumors recurred in three of those who were treated with conservative surgery (15.0%); one patient had undergone a marginal resection and the other two had undergone enucleation and curettage. There were no reported recurrences in any patient who underwent radical surgery. The mean follow-up period was 46.4 months.

**Table 1:** Clinical studies of mandibular odontogenic myxoma published between December 1990 and July 2013

**Author**	**Year**	**No. of cases**	**Age**	**Sex**	**Treatment**	**Follow-up period (months)**	**Recurrence**	**Size (mm)**
Miranda et al. [4]	2013	1	55	M	Conservative	12	None	28 × 33
Lahey et al. [5]	2013	1	69	F	Radical	14	None	30 × 30
De Melo et al. [6]	2012	1	20	F	Radical	18	None	NA
Manne et al. [7]	2012	1	19	M	Radical	30	None	NA
Albanese et al. [8]	2012	1	25	F	Conservative	6	None	21.2 × 47.6
Mauro et al. [9]	2012	1	6	M	Conservative	6	None	NA
Kansy et al. [10]	2012	1/4	33	F	Conservative	180	Recurrence	NA
Boffano et al. [2]	2011	8/10	20	M	Conservative	42	None	20
			38	M	Conservative	38	None	25
			42	F	Conservative	40	None	30
			36	F	Radical	12	None	40
			31	F	Radical	108	None	40
			55	M	Radical	165	None	45
			50	M	Radical	15	None	50
			41	M	Radical	112	None	50
Lin et al. [11]	2010	1	25	F	Conservative	24	None	NA
Reddy et al. [12]	2010	1/2	26	F	Radical	24	None	NA
Rajasekher et al. [13]	2009	1	17	F	Conservative	48	None	70 × 30
Leiser et al. [14]	2009	1/3	52	M	Radical	60	None	NA
Landes et al. [15]	2008	1	14	M	Radical	30	None	NA
Li et al. [16]	2006	12/25	40	F	Radical	5	None	NA
			66	F	Radical	24	None	NA
			32	M	Conservative	84	None	NA
			20	M	Radical	12	None	NA
			17	F	Radical	30	None	NA
			46	M	Radical	48	None	NA
			12	M	Radical	36	None	NA
			36	F	Radical	3	None	NA
			7	M	Conservative	84	None	NA
			22	F	Radical	84	None	NA
			37	M	Conservative	132	None	NA
			32	F	Radical	44	None	NA
Sharma et al. [17]	2003	1	23	F	Radical	48	None	NA
Kimura et al. [18]	2001	1	20	F	Radical	24	None	NA
Sumi et al. [1]	2000	1	48	M	Conservative	22	None	15 × 25 × 70
Shimoyama et al. [19]	2000	1	51	M	Conservative	24	None	30 × 28
Lo Muzio et al. [20]	1996	6/10	28	M	Conservative	24	Recurrence	NA
			16	F	Conservative	31	None	NA
			17	M	Radical	60	None	NA
			65	F	Conservative	84	None	NA
			22	F	Conservative	48	Recurrence	NA
			21	M	Conservative	24	None	NA
Schneck et al. [21]		1/2	24	F	Radical	60	None	NA
Bucci et al. [22]	1993	1	28	M	Conservative	24	None	30 × 40

*Abbreviations*: *M* male, *F* female, *NA* not available.

## Discussion

Thoma and Goldman first described odontogenic myxoma of the jaw in 1947 [23]. Odontogenic myxoma is generally regarded as a rare benign tumor that occurs in tooth-bearing areas of the mandible and maxilla, and is characterized by its slow growth and bony invasions, resulting in painless facial deformity. Its radiological appearance is of a “soap bubble” or “tennis racquet strings” [24]. Our patient reported no symptoms in the right mandibular area; however, panoramic radiography revealed an extensive radiolucent and multilocular area with imprecise borders that extended from the right posterior mandibular body to the area around the root of tooth #46.

Initial treatment for odontogenic myxoma is surgery, but there appears to be some controversy about the best approach to take and evidence-based management guidelines have not been established. To investigate the influence of a conservative or radical surgical approach to odontogenic myxoma, we undertook a search of the PubMed database in July 2013 using the search term “odontogenic myxoma” that identified 211 studies published between December 1990 and July 2013 (Figure 5). Articles for which full text was not available or with missing data were excluded. The second step involved filtering the remaining 181 studies by patient age and sex, tumor location (maxilla/mandible), treatment approach (conservative/radical), recurrence, and follow-up period. Conservative treatment was defined as enucleation, curettage, and marginal resection; radical treatment was defined as segmental or block resection, and hemimandibulectomy requiring reconstruction. After this second step, 45 articles were identified, of which 21 (reporting 44 cases of mandibular disease) were selected for analysis. Details of the 44 cases are given in Table 1. The mean age of patients was 31.9 years, and 22 (50.0%) were male. Twenty of the 44 cases (45.5%) were managed by conservative surgical techniques, the remainder underwent radical treatment. Tumors recurred in three of those who were treated with conservative surgery (15.0%); one patient had undergone a marginal resection and the other two had undergone enucleation and curettage. There were no reported recurrences in any patient who underwent radical surgery. The mean follow-up period was 46.4 months. Most oral-maxillofacial surgeons consider relevant characteristics to be the location of the tumor, its size and type, the patient’s age, sex and clinical characteristics, and the risk of recurrence.

Conservative treatments have several advantages over more radical treatments, such as segmental or block resection, and hemimandibulectomy with reconstruction surgery. Conservative treatments are substantially less invasive, can be achieved by means of an intraoral surgical approach, preserve function and aesthetics, have a shorter hospitalization time, and are more cost-effective [3]. Nonetheless, the risk of recurrence after more conservative surgery is greater as the myxoma is not encapsulated and its myxomatous tissue infiltrates the surrounding bony tissue without causing immediate destruction [3]. Therefore, complete surgical removal can be challenging, which may explain the high recurrence rates (10–30%) after conservative surgical treatment for odontogenic myxoma [20].

Differences in recurrence rate appear to be entirely accounted for by treatment approach: the rate after simple enucleation and curettage has been reported to be as high as 25% [14]. The main reason for recurrence is thought to be incomplete removal rather than the intrinsic biological behavior of the tumor [25]. Several investigators have recommended that tumor size should determine whether a radical or more conservative surgical approach should be adopted [20]. Boffano *et al*. suggested that conservative treatment by enucleation and curettage is recommended when the diameter of an odontogenic myxoma is less than 3 cm, whereas a segmental resection with immediate reconstruction is preferred in patients with larger tumors [2]. Our literature review found that recurrence was only reported after conservative treatment (Table 1). Kancy *et al*. reported a polycystic lesion in the right retromolar mandible, for which they performed a resection with preservation of mandibular continuity, filling the defect with an autologous bone graft from the iliac crest a week later [10]. Nevertheless, 5 years after surgery, a recurrence was detected. Lo Muzio *et al.* described two cases of recurrence after resection of large unilocular radiolucent lesions causing tooth displacement and nonhomogeneous bone reabsorption with extrusion of the third molar; both after enucleation and curettage [20]. Recently, Zanetti *et al*. strongly suggested conservative treatment should involve enucleation of the lesion with a wide curettage of normal tissue or a generous amount of apparently uninvolved surrounding tissue, or even peripheral osteotomy, as this has the advantage of preserving vital structures and maintaining oral function [26]. They also reported that this technique could be used to treat odontogenic myxoma should it recur after more conservative surgery [26]. In our case, the tumor was relatively large (approximately 30 × 15 × 40 mm), but even though its diameter was greater than 3 cm, we chose a more conservative approach after obtaining informed consent from the patient. As odontogenic myxoma is so rare it is not possible for a single center to accumulate sufficient expertise to examine whether a more conservative approach is also suitable for larger tumors. Indeed, the tumor in our patient was >3 cm diameter and there has been no recurrence after enucleation and wide curettage of normal surrounding tissue. If a patient should ultimately develop a recurrence having being treated according to a more conservative strategy, careful consideration should be given to the subsequent treatment. Although there is little evidence upon which to base management decisions, we recommend that further, radical surgery is warranted after recurrence of conservatively-treated odontogenic myxoma, a view that concurs with reports of three cases in the literature [10,20].

A follow-up period is clearly also necessary. It has been recommended that patients should be followed closely for at least the first 2 years after surgery, which represents the period during which the neoplasm is most likely to recur [20]. Rocha *et al.* suggested that 5 years of surveillance is needed to confirm successful excision, but that ideally follow-up should be maintained indefinitely [3]. Although our patient underwent surgery more than 10 years ago, there have been no clinical or radiologic signs of recurrence.

## Conclusions

The successful clinical management of this case and our systematic review of the literature should help inform treatment decisions for odontogenic myxoma, to minimize the risk of recurrence while adopting a less invasive surgical approach whenever possible.

## Consent

Written informed consent was obtained from the patient for publication of this Case Report and any accompanying images. A copy of the written consent is available for review by the Editor-in-Chief of this journal.

### Ethical approval

Conduct of this study conformed to the Declaration of Helsinki, and was approved by our institutional ethical committee. The patient was fully informed of the procedures and possible risks of the study, and freely gave written consent.

## Competing interests

The authors declare that they have no competing interests.

## Authors’ contributions

All authors were involved in the direct diagnosis of the reported patient, were involved in the preparation of the manuscript, and read and approved the final version.
